# The impact of left atrial voltage abnormality on ablation outcomes in paroxysmal atrial fibrillation and its pre-procedural predictors: an observational retrospective study

**DOI:** 10.1186/s43044-025-00664-w

**Published:** 2025-07-31

**Authors:** Dongsheng Zhao, Yan Dong, Qiushi Chen, Gaoyuan Ge, Nishant Yadav, Di Yang, Fengxiang Zhang

**Affiliations:** 1 Department of Cardiology, The First Affiliated Hospital of Nanjing Medical University, Guangzhou Road 300, Nanjing, 210029 Jiangsu People’s Republic of China; 2 Department of Cardiology, The Second Affiliated Hospital of Nantong University, Shengli Road 666, Nantong, 226000 Jiangsu People’s Republic of China

**Keywords:** Paroxysmal atrial fibrillation, Electro-anatomic mapping, Atrial tachyarrhythmia burden, Outcome

## Abstract

**Background:**

Left atrial (LA) localized voltage abnormality displayed by electro-anatomic mapping (EAM) has been established as a surrogate marker of atrial fibrosis (AF) which predicts post-ablation recurrence. This study investigates preoperative predictors of LA voltage abnormalities and assesses their impact on atrial fibrillation recurrence following catheter ablation in patients with paroxysmal atrial fibrillation (PAF).

**Results:**

Forty-four (25.6%) patients had LA voltage abnormality which independently predicted post-ablation recurrence (HR 2.85, 95%CI 1.20–6.78, *p* = 0.02). Larger left atrial diameter (LAD) (OR 1.24 per 1 mm, 95%CI 1.02–1.50, *p* = 0.03) and higher atrial tachyarrhythmia (ATA) burden (OR 1.02 per 1%, 95%CI 1.00–1.04, *p* = 0.03) independently correlates with LA voltage abnormality with an ideal combined diagnostic efficiency (AUC 0.80, sensitivity 79.3%, specificity 70.7%, 95% CI 0.70–0.89, *p* < 0.01).

**Conclusions:**

LA substrate abnormality, even moderate, is an independent risk factor for PAF post-ablation recurrence which can be predicted pre-procedure by LAD and ATA burden.

## Background

While circumferential pulmonary vein isolation (CPVI) is highly efficacious for most PAF, a significant portion of patients still have recurrence necessitating repeat procedures [1, 2]. AF can induce atrial structural remodeling, causing AF to further induce AF [3, 4]. Atrial fibrosis progresses with increasing AF burden [3, 5] and predicts ablation failure [6]. LA low-voltage area (LVA) detected by EAM as a surrogate of fibrosis has been cross-validated by cardiac magnetic resonance imaging (CMRI) [7, 8]. Previous studies from our center had verified that AF progression was associated with declining LA subendocardial voltage [9] and the moderate LA voltage abnormality was associated with recurrence after CPVI in persistent AF patients [10]. Referring PAF, the association of the ATA burden and the LA subendocardial voltage and their prediction efficacy of post-ablation recurrence has been rarely researched up to now. We aimed to investigate the impact of LA voltage abnormality on ablation outcomes in PAF patients and its pre-procedural predictors including the ATA burden.

## Methods

### Study population and ATA burden assessment

This is a single-center retrospective observational cohort study conducted between January 2020 and October 2022 at the first affiliated hospital of Nanjing Medical University in Nanjing, China. A total of 172 consecutive patients with symptomatic, drug-refractory PAF, who underwent high-density LA EAM before ablation, were enrolled in this study. Patients with a LA mapping number of < 1000 points, significant structural heart disease (SHD), left ventricular ejection fraction (LVEF) < 40%, previous myocardial infarction, severe renal impairment (including dialysis), previous AF ablation, and patients who had undergone open heart surgery were excluded from this study. ATA burden at baseline was defined as the percentage of monitoring time spent in ATA [total number of hours spent in AF, atrial tachycardia (AT), or atrial flutter (AFL) sustaining ≥ 30 s/total number of recording hours]. We collected the ATA burden from the Holter performed in the 1 week before ablation and in the absence of antiarrhythmic medication.

### EAM protocol and ablation procedure

**Fig. 1:**
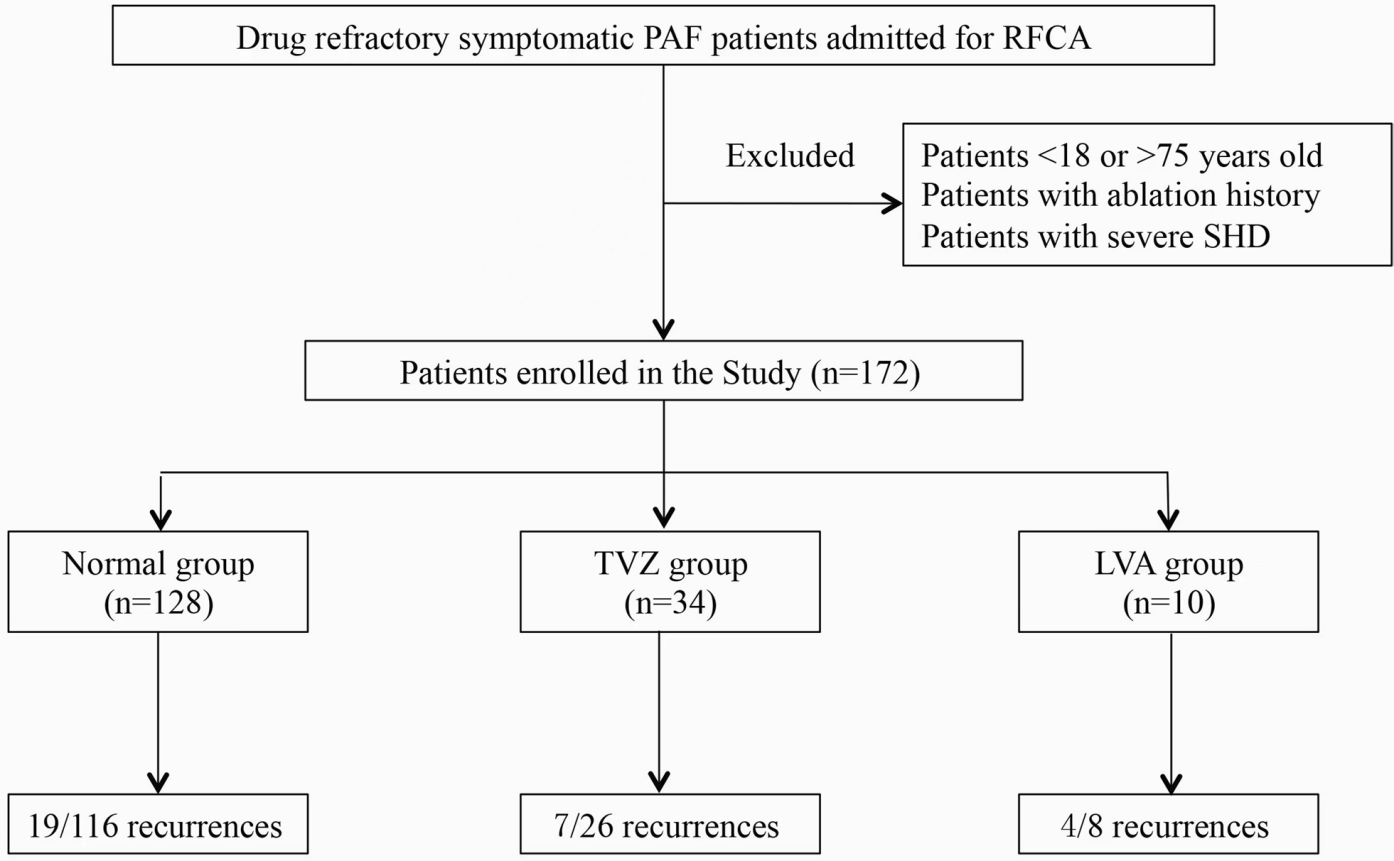
Patients enrollment and follow-up

The operation was performed using a three-dimensional EAM system (CARTO 3, V6.0 Biosense Webster, Diamond Bar, USA). After written informed consents were obtained from all patients, antiarrhythmic drugs were discontinued at least five half-lives before the procedure. A decapolar and a quadripolar electrode catheter was positioned in the coronary sinus and superior vena cava, respectively, via the femoral vein. An ablation catheter (Thermocool SmartTouch, Biosense Webster, Diamond Bar, CA) and a mapping catheter (Pentaray, Biosense Webster, Diamond Bar, CA) were introduced into the LA through two trans-septal sheaths. Intravenous heparin was administered to achieve an activated clotting time of 300–350 s. In all cases, LA geometry and detailed bipolar voltage maps were created simultaneously using the mapping catheter in fast-anatomical mapping mode during sinus rhythm before ablation. Mapping was performed with an equal distribution of points using a fill threshold of 7 mm excluding PV ostia with the target of > 1000 points. The electrode–tissue contact was evaluated through the fluoroscopic motion, deformation of the catheter caused by contact with the wall, catheter icon-to-surface feature of the mapping system, and the presence of sharp near-field electrograms. LVA was defined as a bipolar peak-to-peak voltage amplitude of 0.1–0.4 mV and the transitional voltage zone (TVZ) 0.4–1.3 mV [9]. The extent was defined as the total LVA and TVZ areas divided by the entire LA surface area. Patients were considered to have voltage abnormality if LVA/TVZ extended over more than 5% of the entire LA surface area in accordance with previous studies [11]. After the shell building and voltage mapping were accomplished, we set the voltage bar range as 0.1–0.4 mV; if the result was positive, this patient belongs to the LVA group, then, we set the range as 0.4–1.3 mV; if the result was positive, this patient belongs to the TVZ group, otherwise, it belongs to the normal group (no areas of voltage < 1.3 mV) (Fig. 1). A CPVI plus trigger-based ablation procedure was performed using radiofrequency energy alone and the recommended energy setting was 35–40 W, which had been reported in our previous study [12].

### Follow-up

Follow-up visits were performed at the 1st, 3rd, 6th, and 12th and every 6 months thereafter. 24-h Holter was performed at the 1st, 3rd, and 6th month, while 7-day Holter was arranged on the 12th month visit. When recurrence was suspected on the basis of symptoms reporting palpitations, shortness of breath, chest pain, fatigue, or any combination of the above, an additional 7-day Holter monitoring was performed. A 3-month blanking period was used after the ablation with uninterrupted antiarrhythmic drugs and anticoagulation. After this period, the occurrence of ATA lasting more than 30 s was defined as the AF recurrence.

### Statistical analysis

Categorical variables are summarized as numbers and percentages and continuous variables as mean ± standard deviation (SD) or median and quartile. The Shapiro–Wilk test was performed to confirm that the data were normally distributed. Clinical characteristics expressed as categorical variables were compared using the Chi-square test. And the ANOVA test or Kruskal–Wallis test was used for continuous variables depending on the distribution pattern. To test correlation between the two variables, Pearson’s correlation coefficient (Pearson’s r) was calculated. The multivariate logistic regression analysis was used to determine independent predictors of TVZ including all the variables with a *p* value < 0.05 at the intergroup comparison. And the diagnostic power and the optimal cutoff point were identified using receiver operator characteristic (ROC) analysis. The follow-up period was defined as the period from the date of the procedure to the date of the first AF recurrence or end of follow-up. The HR was calculated by univariate and multivariate Cox proportional regression models to evaluate the prognostic power of variables in predicting the AF recurrence. In the multivariable models, we entered all clinical covariates with a *p* value < 0.20 at univariate analysis, excluding those with significant correlations with other covariates. The estimated event-free survival probabilities were calculated using the Kaplan–Meier analysis, and log-rank statistics was used for group comparisons. All analyses were performed using SPSS software (version 13.0, SPSS Inc., Chicago, IL), and the p value reported was two-sided and value of less than 0.05 was considered statistically significant.

## Results

### Patients and LA substrate characteristics

The flow diagram of patients’ enrollment is shown in Fig. 1. A total of 172 patients were included: 117 males and 55 females with a mean age of 59.5 ± 9.9 years. LA voltage abnormality was identified in 44 (25.6%) including TVZ in 34 and LVA in 10 with the median extent of LA surface area involvement being 11.8% and 11.2%, respectively.

### Clinical and mapping characteristics

**Fig. 2:**
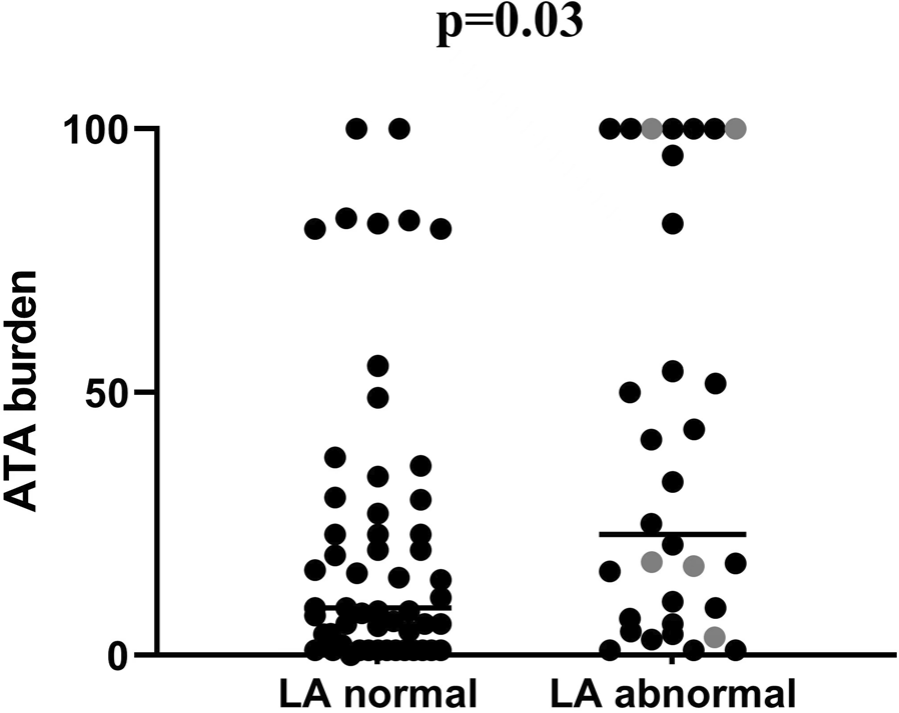
ATA burden comparison between the two groups. ATA: atrial tachyarrhythmia

The baseline clinical and EAM characteristics are shown in Table 1. The patients with LA voltage abnormality were older with more females and larger LAD. BMI and the course of AF were not significantly different among the three groups. ATA burden was significantly higher in the TVZ and LVA group than in the normal group (median 29.0% and 17.7% vs. 8.7%) (*p* = 0.03) (Fig. 2). In addition, there was a slight correlation between ATA burden and LA TVZ extent: Pearson R = 0.31, *p* = 0.04. The multivariate logistic regression analysis showed that LAD (OR 1.24 per 1 mm, 95%CI 1.02–1.50, *p* = 0.03) and ATA burden (OR 1.02 per 1%, 95%CI 1.00–1.04, *p* = 0.03) were independently positively correlated with LA voltage abnormality (Table 2).

**Table 1:** The baseline clinical and EAM characteristics among three groups

	Normal group (*n* = 128)	TVZ group (*n* = 34)	LVA group (*n* = 10)	*p* value
*General characteristics*				
Age (year)	58.4 ± 9.2	61.4 ± 10.8	65.8 ± 13.3	*0.03*
Female (%)	31 (24.2%)	17 (50.0%)	7 (70.0%)	< *0.01*
BMI	24.5 ± 2.7	25.2 ± 2.9	25.4 ± 3.1	0.39
Disease course (month)	24 (6, 36)	19 (5, 72)	18 (10, 70)	0.96
ATA burden (%)^*^	8.7 (1.0, 29.0)	29.0 (4.9, 53.4)	17.7	*0.03*
Pro-BNP (ng/L)	100 (60, 281)	160 (60.0, 282.0)	1237 (889, 1530)	*0.04*
CHA_2_DS_2_-VASc score	1 (0, 2)	1 (1, 3)	2 (2, 3)	< *0.01*
HAS-BLED score	0 (0, 1)	1 (0, 1)	1 (1, 2)	< *0.01*
*Complication (%)*				
HTN	57 (45.6%)	17( 50.0%)	8 (80.0%)	0.10
Diabetes	13 (10.2%)	4 (11.8%)	1 (10.0%)	0.97
CHD	12 (9.4%)	2 (5.9%)	2 (20.0%)	0.45
Stroke	4 (3.1%)	2 (4.8%)	0 (0.0%)	0.54
*Medication(%)*				
β receptor blocker	36 (28.3%)	10 (29.4%)	5 (50.0%)	0.38
ACEI/ARB	20 (15.7%)	10 (29.4%)	4 (40.0%)	0.71
CCB	18 (14.1%)	10 (29.4%)	4 (40.0%)	*0.04*
Statin	24 (18.9%)	12 (35.3%)	3 (30.0%)	0.13
*Transthoracic echocardiographic*				
LAD (mm)	37.5 ± 3.5	39.8 ± 4.2	40.8 ± 3.1	< *0.01*
RAD (mm)	34.2 ± 3.7	35.4 ± 3.8	36.4 ± 3.6	0.07
LVEF (%)	63.3 (2.7)	63.0 (2.6)	63.0 (4.7)	0.48
*EAM(%)*				
PV trigger	38 (38.0%)	5 (41.7%)	3 (60%)	0.89
Non-PV trigger	25 (25.0%)	3 (25.0%)	2 (40%)	/
Unmapped	33 (33.0%)	4 (29.4%)	1 (20%)	/
Multiple triggers	4 (4.0%)	0 (0%)	0 (0%)	/
Combined with SVT	14 (10.9%)	6 (17.6%)	2 (20.0%)	0.48

*p* < 0.05 was marked as italics

EAM: electro-anatomic mapping; TVZ: transitional voltage zone (0.4–1.3 mV); LVA: low voltage (0.1–0.4 mV); BMI: body mass index; ATA: atrial tachyarrhythmia; HTN: hypertension; CHD: coronary heart disease; ACEI/ARB: angiotensin-converting enzyme inhibitors/angiotensin receptor blockers; CCB: calcium channel blockers; LAD: left atrium diameter; RAD: right atrium diameter; LVEF: left ventricular ejection fraction; PV: pulmonary vein; SVT: supraventricular tachycardia

^*^ 58 cases in the normal group, 26 cases in TVZ group, and 5 in LVA group

**Table 2:** The multivariate logistic regression analysis of the presence of LA voltage abnormality

Parameter	OR (95% CI)	*p* value
Age	1.02 (0.96,1.08)	0.53
Sex (female)	2.80 (0.79, 10.0)	0.11
**ATA burden (%)**	**1.02 (1.00, 1.04)**	***0.03***
proBNP (ng/L)	1.00 (0.99, 1.00)	0.68
**LAD (mm)**	**1.24 (1.02, 1.50)**	***0.03***
CCB	1.22 (0.23, 6.67)	0.81

The OR > 1 with *p* < 0.05 was marked as bold and bold italics

ATA: atrial tachyarrhythmia; LA: left atrium; LAD: left atrium diameter; CCB: calcium channel blockers

### ROC curve analysis to predict LA voltage abnormality

**Fig. 3:**
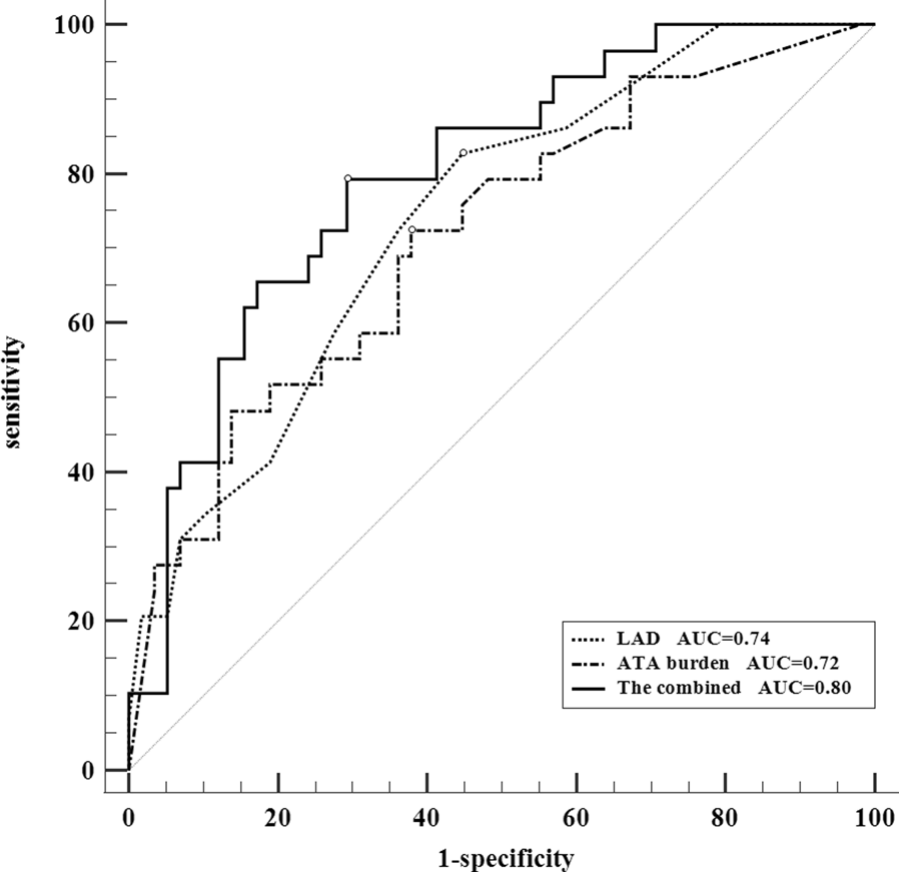
The diagnostic efficacies of LAD, ATA burden, and their combination on LA voltage abnormality. LAD: left atrium diameter; ATA: atrial tachyarrhythmia; LA: left atrium

We conducted ROC curve analysis to evaluate the diagnostic value of the two independently related parameters (LAD/ATA burden) for LA voltage abnormality. Their combined diagnostic efficacy (AUC: 0.80, sensitivity: 79.3%, specificity: 70.7%, 95% CI 0.70–0.89, *p* < 0.01) was higher (*p* = 0.01) than separately: LAD (AUC: 0.71, sensitivity: 78.0%, specificity: 55.1%, 95% CI 0.64–0.78, *p* < 0.01), ATA burden (AUC 0.69, sensitivity 46.7%, specificity 84.7%, 95% CI 0.59–0.79, *p* < 0.01) (Fig. 3).

### LA voltage abnormality and the ablation outcome

**Fig. 4:**
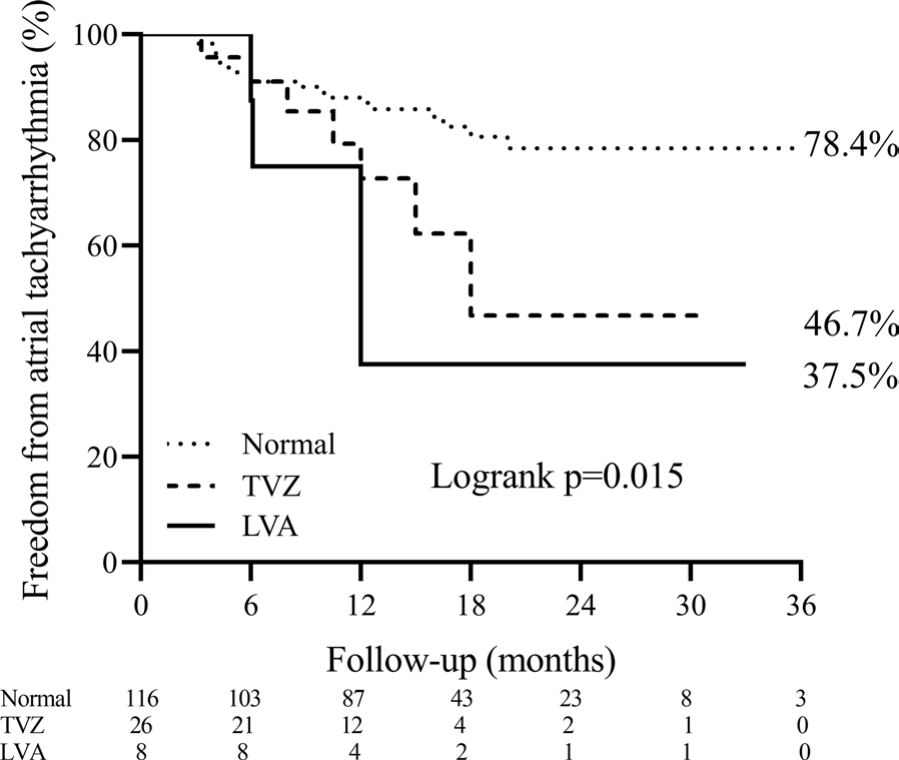
Kaplan–Meier curves of freedom from any atrial tachyarrhythmia (AF/AFL/AT) after a single ablation procedure, stratified by LA substrate mapping characteristics. AF: atrial fibrillation; AFL: atrial flutter; AT: atrial tachycardia; LA: left atrium; TVZ: transitional voltage zone; LVA: low-voltage area

After an average follow-up of 14.6 ± 8.6 months, the patients with LA voltage abnormality had significantly lower single-procedure arrhythmia-free survival. The Kaplan–Meier survival curve is shown in Fig. 4. In multivariate COX analysis, only the presence of LA voltage abnormality was significantly associated with AF recurrence after a single ablation (Table 3). Overall 30 patients had AF recurrence, 19 in the normal group, 7 in the TVZ group, and 4 in the LVA group, whereas 3, 0, and 2 AT episodes occurred in the respective group, without significant difference.

**Table 3:** Univariable and multivariable analysis of single-procedure arrhythmia-free survival

Parameter	Univariate		Multivariate	
	HR (95% CI)	*p* value	HR (95% CI)	*p* value
ACEI/ARB	1.92 (0.81, 4.54)	0.14	1.69 (0.67, 4.35)	0.27
LAD	1.09 (0.98, 1.21)	0.10	1.06 (0.95, 1.18)	0.26
LA voltage abnormity	3.40 (1.53, 7.56)	< 0.01	2.85 (1.20, 6.78)	*0.02*
TVZ	4.35 (1.41, 12.50)	0.01	1.70 (0.66, 4.36)	0.05
LVA	1.69 (0.50, 6.25)	0.41	–	–

HR: hazard ratio; ACEI/ARB: angiotensin-converting enzyme inhibitors/ angiotensin receptor blockers; LAD: left atrium diameter; TVZ: transitional voltage zone (0.4–1.3 mV); LVA: low-voltage area (0.1–0.4 mV)

## Discussion

### Main findings

This retrospective cohort study, which stratified PAF patients undergoing RFCA according to LA voltage mapping findings and compared their clinical profiles with procedural outcomes, yielded two principal observations: (i) The presence of LA voltage abnormality is a crucial risk factors of AF recurrence after RFCA, and (ii) LAD and ATA burden is independently positively correlated with LA voltage abnormality and has an ideal combined diagnostic efficiency (AUC 0.80, sensitivity 79.3%, specificity 70.7%) optimal than separately.

### Atrial remodeling progressing in PAF

Atrial fibrosis plays a central role in the maintenance and perpetuation of AF [13, 14] and progresses along with the AF type [3, 9]. In PAF itself, the presence of LA LVA varies between 10 and 34% [15–17]; this rate was 4.4% in our study. The discrepancy may come from the patients’ selection, LVA definition or the mapping instrument. Definition of PAF based on episodes lasting less than 7 days encompasses a heterogeneous group in terms of substrate remodeling and prognosis [18]. To date, little attention has been paid to the atrial remodeling progression within PAF itself. A goat AF model revealed that a longer duration of AF results in significantly greater endomysial fibrosis (6 months vs. 3 weeks) [5]. Few clinical studies [19, 20] had shown that atrial structural remodeling was progressing along with the increase in AF burden in PAF. Recently, Strisciuglio et al. [15] showed that PAF patients with high ATA burden (≥ 9.3%) have altered LA mechanical properties, reflected by larger size and impaired function. In agreement with the previous evidence, our result shows that PAF patients with LA substrate abnormality had significantly higher ATA burden (median 29.0%) and it is positively related to the extent.

### Atrial remodeling and prognosis

It is well known that AF is strongly associated with an increased risk of thromboembolic events, acute decompensated heart failure (HF), and other cardiovascular events. But now, the growing body of evidence suggests that atrial cardiomyopathy (fibrosis) may be the key, rather than the AF rhythm itself. Through the follow-up study up to 5 years of a large AF cohort, King et al. [21] had found that there was a strong and graded association between severity of LA fibrosis, defined as the quantity of late gadolinium enhancement (LGE) observed on cardiac magnetic resonance imaging (CMRI), and the incidence of MACCE (major adverse cardiovascular and cerebrovascular events) which adds to the other data [22, 23] indicates that LA LGE severity is an independent risk factor for stroke, rather than AF rhythm alone. In our present study, we also observed that the LA voltage abnormality was related with higher CHA2DS2-VASc score and more cerebrovascular events. Atrial fibrosis was also reported to be an independent predictor of failure of LVEF improvement after ablation [24] and new-onset or aggravated heart failure [21, 25]. In today's era of ablation, plenty of studies have identified LA fibrosis also as the key factor of arrhythmia recurrences based on both CMRI [7, 26] and electrical remodeling [1, 2, 11, 27]. CPVI alone strategy for nonparoxysmal atrial fibrillation (NPAF) patients without LVA was enough, with the arrhythmia-free survival comparable with PAF [10, 27], whereas the ablation outcome of PAF patients with LVA is no better than NPAF. Vlachos K et al. [2] demonstrated that the existence of LVA (< 0.4 mV) more than 10% of the total LA surface area predicted arrhythmia recurrence following CPVI for PAF with the success rate less than 60% during 20-month follow-up. Ahmed-Jushuf, F et al. [27] found that PAF patients with LVA (0.2–0.5mv) had only 38% 12-month CPVI arrhythmia-free survival, significantly less than the 61% in persistent AF patients without LVA (93% had CPVI alone). The recurrence in patients with LVA compared to those without was more likely to be AT rather than AF, which was not seen in our study. Perhaps due to small sample size and short time of monitoring, we and a recent study [15] did not find a direct relation between ATA burden and ablation outcome. Andrade, J. G. et al. [28] found that “high-burden PAF” classified by device (implantable cardiac monitor) rather than clinical definition had more recurrent AF post-ablation as compared to the “low-burden PAF”. They also found that patients with AF episodes limited to less than 24 continuous hours had a significantly lower incidence of arrhythmia recurrence following ablation [29].

### Risk factors of LA voltage abnormality in PAF

Easy, reliable prior identifying arrhythmogenic substrate of AF is very helpful for ablation strategy planning and technology selection. For example, in the case of low probability of LA substrate presence, the interventionalist could choose single-shot technologies such as cryoballoon where only CPVI is enough. Different scores calculated using baseline patients' characteristics have been developed to predict LVA. The APPLE score (one point for age > 65 years, persistent AF, impaired eGFR < 60 mL/min/1.73 m^2^, LAD ≥ 43 mm, LVEF < 50%, respectively), initially developed for the prediction of AF recurrences after catheter ablation, has been recently identified as a predictor of LVA (AUC 0.711–0.720) [30–32]. Similarly, the DR-FLASH score (one point for diabetes mellitus, renal dysfunction, persistent form of AF, LAD > 45 mm, age > 65 years, female sex, and hypertension respectively) has also been proved to be effective in predicting the presence of LVA (AUC 0.766–0.819) [16, 30, 32]. However, their method is cumbersome. In this study, we found two handy parameters “ATA burden ≥ 37.6%” and “LAD ≥ 37 mm” be independently correlated with the presence of LA substrate abnormality with a satisfactory combined diagnosis efficacy (AUC 0.80, sensitivity 79.3%, specificity 70.7%) higher than the above in PAF patients without SHD or severe renal impairment.

### Limitation

This study is limited by its observational, retrospective design in a small single-center cohort. The level of operators executing mapping and ablation was not homogeneous, and the process was not under any supervision. Furthermore, only the minority of the ATA episodes had been captured by 24–72 h Holter but not long-term monitoring such as one-week Holter or loop recorders. Also, the pattern of ATA episodes (many, short episodes or fewer, longer episodes) and the substrate of right atrium were not available and analyzable. Finally, the distribution of the voltage abnormality and its relation with AF triggers were not reported.

## Conclusion

LA voltage abnormality, even moderate, is independently related to post-ablation recurrence in PAF patients. It can be predicted pre-procedure by LA dilation (LAD ≥ 37 mm) and high ATA burden (≥ 37.6%).

## Data Availability

All data generated or analyzed during this study have not been published but can be obtained by contacting the authors.

## Abbreviations

LA: Left atrial
EAM: Electro-anatomic mapping
AF: Atrial fibrosis
PAF: Paroxysmal atrial fibrillation
NPAF: Nonparoxysmal atrial fibrillation
LAD: Left atrial diameter
ATA: Atrial tachyarrhythmia
CPVI: Circumferential pulmonary vein isolation
LVA: Low-voltage areas
CMRI: Cardiac magnetic resonance imaging
AT: Atrial tachycardia
AFL: Atrial flutter
TVZ: Transitional voltage zone
